# Extranodal natural killer/T-cell lymphoma presenting as hypopyon panuveitis: a case report

**DOI:** 10.1186/s12886-022-02277-2

**Published:** 2022-02-01

**Authors:** Nutchaya Sukon, Nattaporn Tesavibul, Pitipol Choopong, Noppakhun Panyayingyong, Sutasinee Boonsopon

**Affiliations:** 1 Department of Ophthalmology, Faculty of Medicine, Siriraj Hospital, Mahidol University, 2 Wanglang Road, Bangkoknoi, Bangkok, 10700 Thailand; 2 Metta Pracharak Hospital (Wat Rai Khing), 52 Moo 2 Rai Khing sub-district, Sampran district, Nakhonpathom, 73210 Thailand

**Keywords:** Case report, Ocular extranodal natural killer/T-cell lymphoma, Panuveitis, Hypopyon uveitis, Ocular masquerade syndrome

## Abstract

**Background:**

Extranodal natural killer/T-cell lymphoma (ENKTL), nasal type, generally affects the orbit by direct extension. It can even rarely present as severe intraocular inflammation mimicking endophthalmitis. Delayed diagnosis and treatment are frequently reported.

**Case presentation:**

A 43-year-old woman presented with 2-month blurred vision in her left eye. Ocular examination revealed hypopyon panuveitis. She was initially diagnosed with endogenous endophthalmitis, which proved irresponsive to antimicrobial therapy. High-dose prednisolone was given afterward, but this failed to stop the development and continuous progression of ocular inflammation. The diagnosis of ENKTL was finally confirmed from the pathological findings of oral ulcers and cervical lymph nodes and chemotherapy was prescribed. After the first cycle of chemotherapy, the patient’s ocular inflammation subsided. Unfortunately, her left eye became phthisis with progressive visual loss within 9 months following the diagnosis.

**Conclusions:**

The diagnosis of intraocular ENKTL is frequently delayed, which can lead to severe problems for treatment as the disease is aggressive with a poor prognosis. It can be found not only in elderly patients but also in a middle-aged individual. In patients with ocular inflammation, a thorough systemic evaluation and histopathological examination of the associated systemic findings is extremely helpful and may reveal the cause of the ocular inflammation, including revealing possible ENKTL.

## Background

Extranodal natural killer/T-cell lymphoma (ENKTL) is an extremely rare subtype of non-Hodgkin’s lymphoma (NHL), believed to be an Epstein–Barr virus-related neoplasm [1–3]. The incidence of NK/T-cell lymphoma (NKTL) is more common in Asian and Latin American populations [2]. Ocular tissue NKTL mostly occurs as nasal ENKTL invasion or dissemination, while intraocular ENKTL has been reported in a few cases [1, 3]. Early ocular manifestations include dacryoadenitis, orbital cellulitis, myositis, and more rarely, uveitis [2, 3]. Only a few studies have reported intraocular NKTL presenting as intermediate uveitis, posterior uveitis, or panuveitis; although some of those cases were initially misdiagnosed as other inflammatory conditions, such as viral-related uveitis or unspecific intraocular inflammation, but failed to respond to antimicrobial and/or corticosteroids therapy [1, 2, 4–8]. In this paper, we report a rare case of ENKTL occurring as uveitis masquerade syndrome [9], or more specifically, hypopyon panuveitis.

## Case presentation

**Fig. 1:**
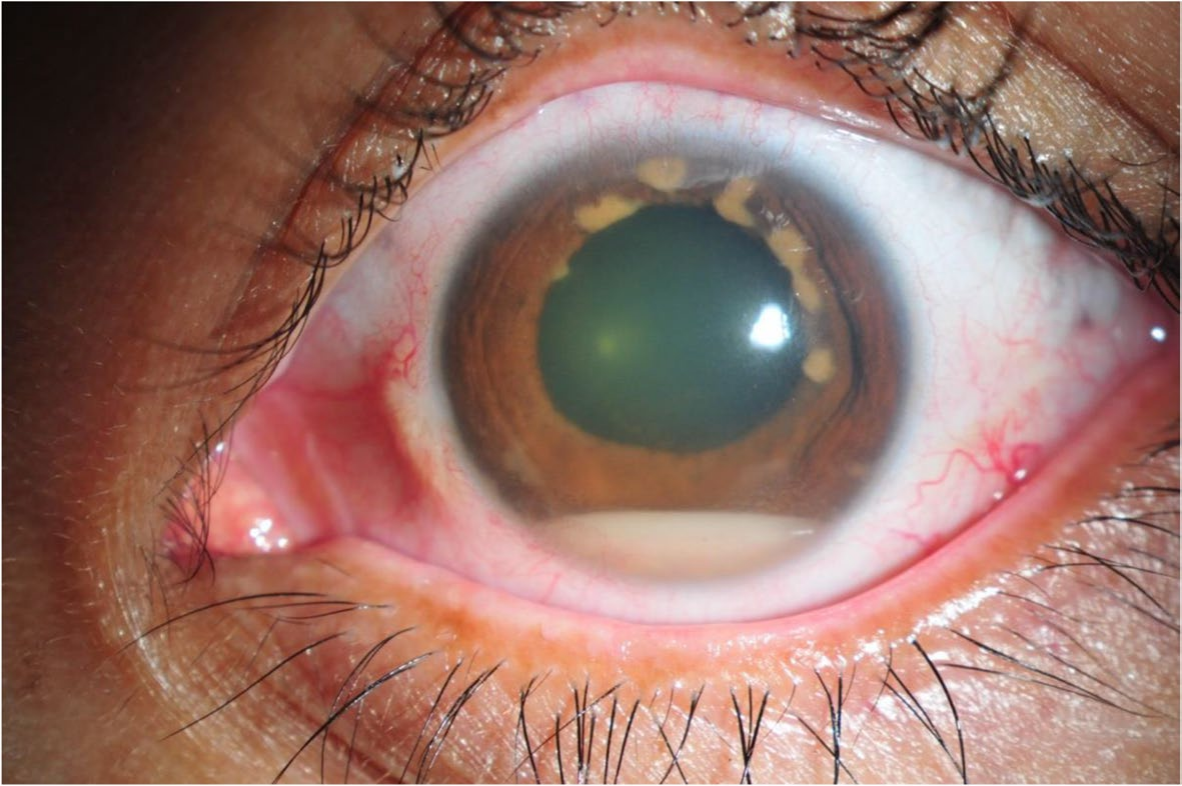
Anterior segment photo of the left eye. The photo shows a subtle conjunctival injection, hypopyon, and multiple hypopigmented iris nodules

**Fig. 2:**
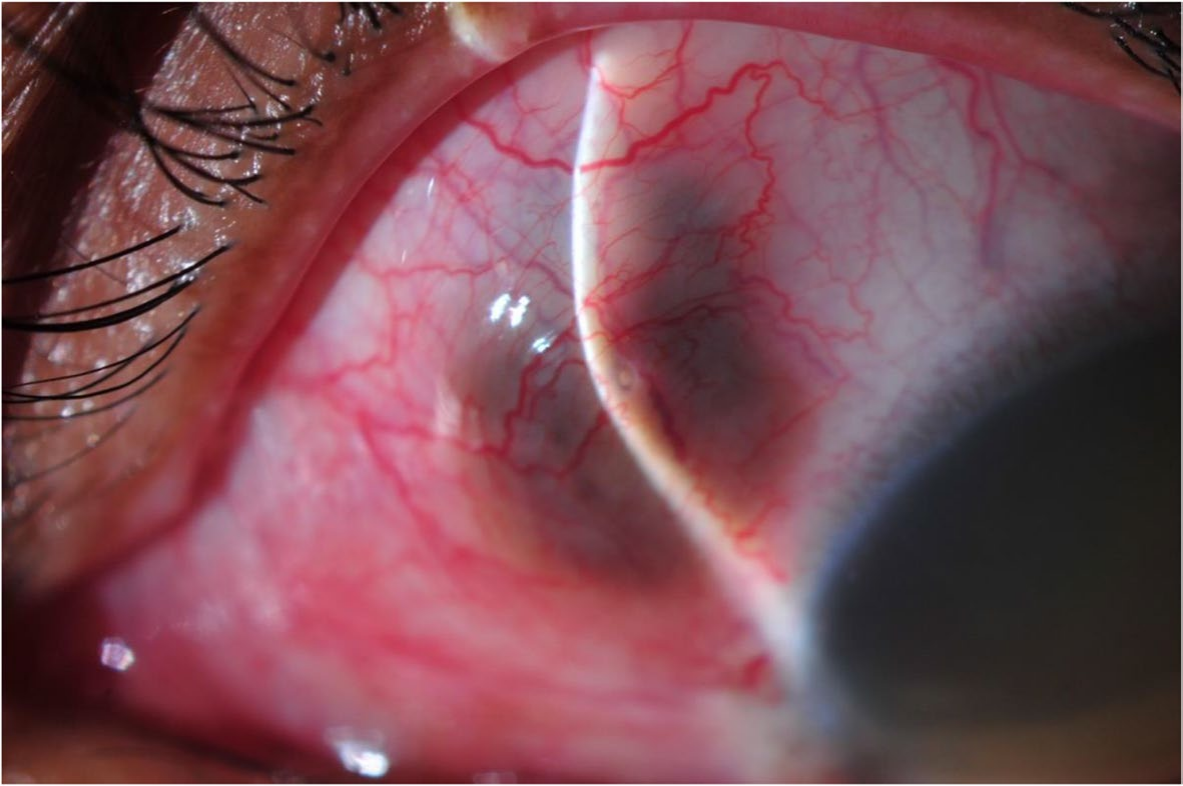
Scleral thinning and prolapsed uveal tissue. Scleral thinning can be seen over the area of the vitreous tapping and intravitreal injection site. A poor-healing needle puncture wound of the sclera is shown adjacent to the slit beam

**Fig. 3:**
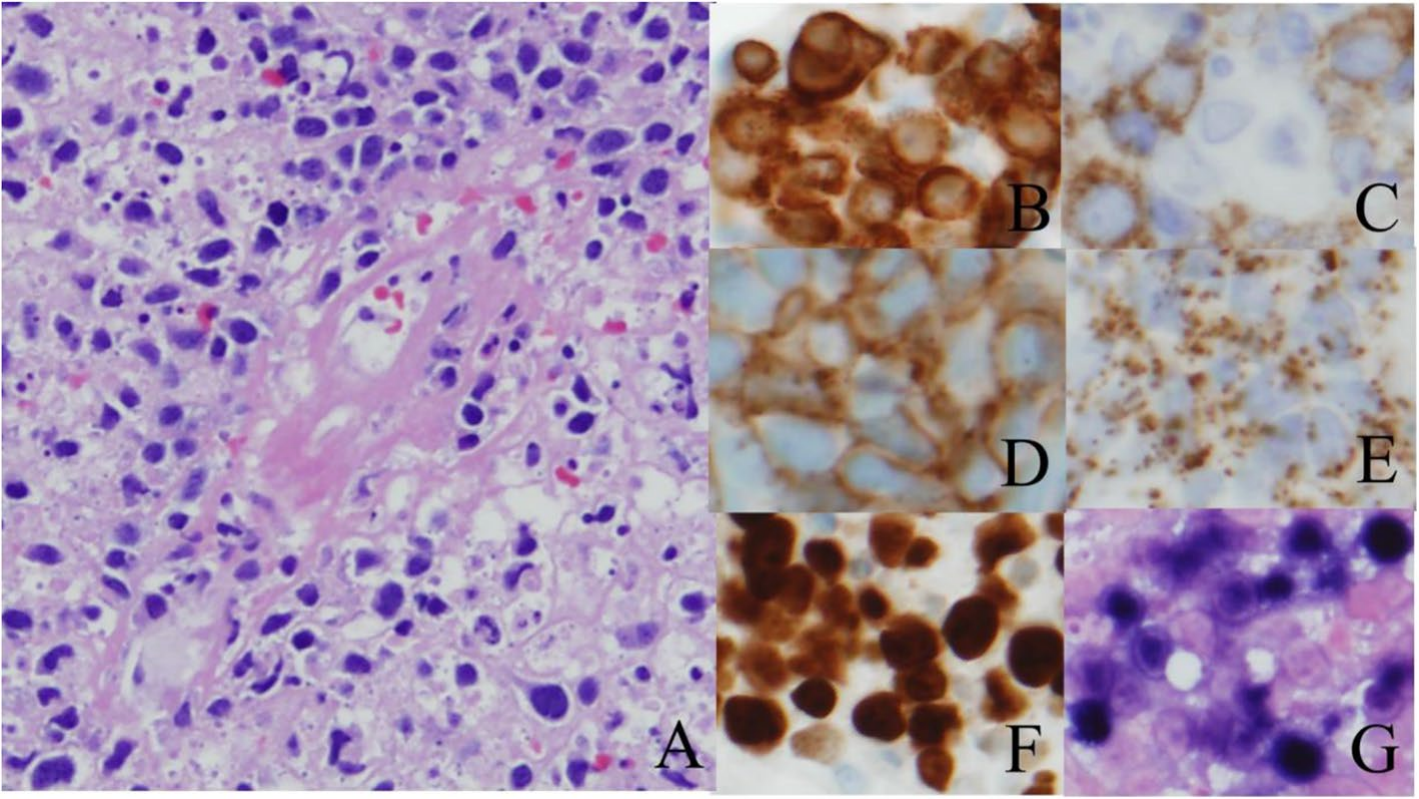
Histopathological reports for the lymph nodes and oral ulcer. Extranodal NK/T-cell lymphoma, nasal type. **A**) Angioinvasion by pleomorphic lymphoid cells. Note one blood vessel in the center with infiltration by lymphoma cells. **B**) Cluster of differentiation (CD) 3 positive lymphoma cells with typical cytoplasmic staining pattern. **C**) Occasional CD30 positive lymphoma cells (membrane staining). **D**) CD56 positive lymphoma cells (membrane staining). **E**) T-cell intracellular antigen-1 positive lymphoma cells, indicative of cytotoxic granules in the cytoplasm of the lymphoma cells. **F**) High proliferation index by Ki-67 in lymphoma cells (nuclear staining). **G**) Epstein-Barr virus-encoded small ribonucleic acid positive lymphoma cells by in situ hybridization (nuclear staining)

**Fig. 4:**
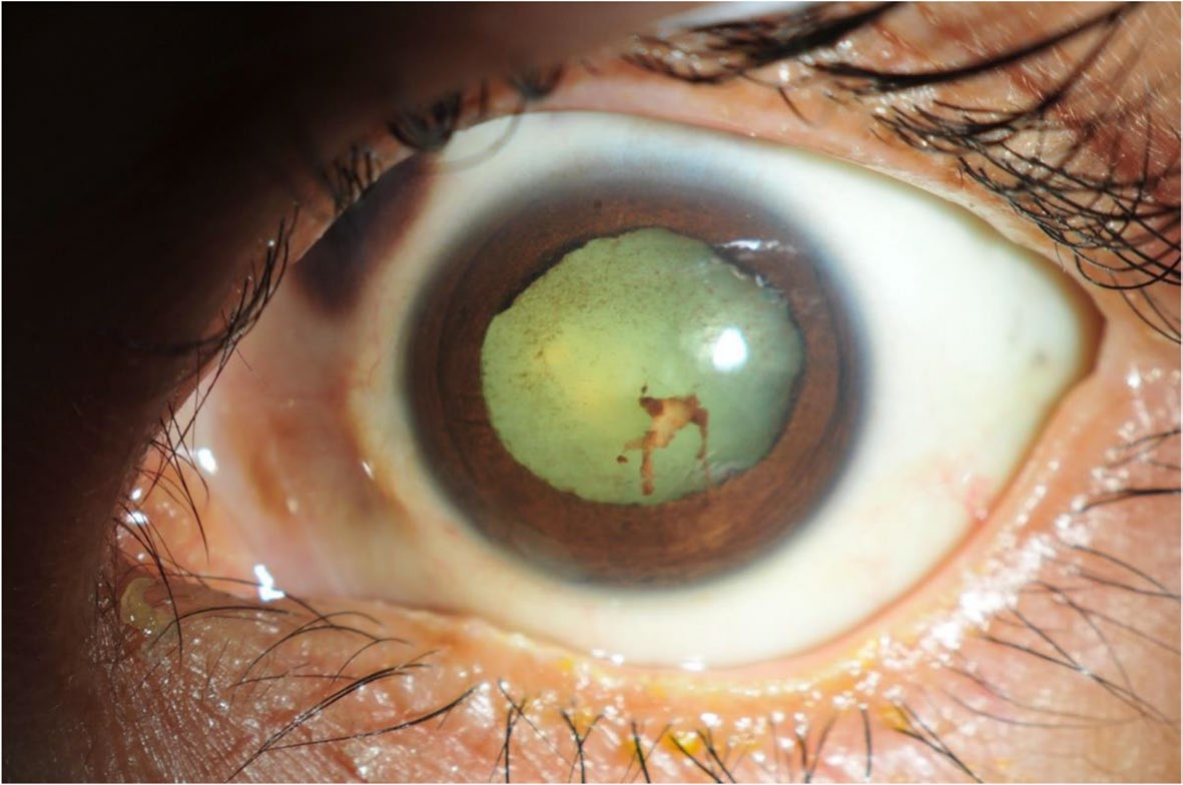
Subsided inflammation. Left eye demonstrating improving inflammation. There was residual fibrin and dispersed pigment over the anterior lens capsule without hypopyon. Posterior synechiae and a complicated cataract were found. Prominent scleral thinning could be obviously seen superonasally

A 43-year-old woman who presented with a 2-month painful blurred vision of her left eye was referred to our uveitis clinic with hypopyon panuveitis in the left eye. Ocular examination revealed a visual acuity (VA) of 6/6 in the right eye and counting fingers at 1-ft in the left eye, intraocular pressure was 12 and 6 mmHg. Her left eye showed 4+ anterior chamber cells with 1.7 mm of hypopyon and a 2+ to 3+ flare with only mild conjunctival injection. There were multiple iris nodules, as shown in Fig. 1. Fundus was obscured. The examination of the right eye was unremarkable. Ultrasonography of the left globe showed a heterogenous vitreous echogenicity, from which endogenous endophthalmitis was initially suspected. She was treated for bacterial endophthalmitis with intravitreal (IVT) vancomycin and ceftazidime injection, and fortified vancomycin and fortified ceftazidime eye drops. Systemic treatment included vancomycin 1-g intravenous infusion every 12 h and ceftazidime 1-g intravenous infusion every 8 h. Relevant investigations to identify the pathogen included vitreous gram stain, potassium hydroxide test, acid-fast bacilli stain, aerobic/anaerobic/mycobacterium culture, and polymerase chain reaction for bacteria/fungus/mycobacteria, which all returned negative results. Also, a hemoculture was performed and displayed no microbial growth. Whole abdominal ultrasound was performed and revealed only a few gallstones, a prominent size of the spleen, and possibly myoma. An echocardiogram showed no evidence of infective endocarditis. A Mantoux test, pathergy test, and anergy test were performed and all returned negative results. The chest radiograph was unremarkable. A plain-film radiograph of the paranasal sinuses showed maxillary sinusitis. Four days later, her clinical presentation worsened, and her VA was recorded as light projection (LP). Atypical mycobacterial infection was considered, for which an injection of IVT amikacin was given. Due to the patient’s worsening clinical status, a blood sample was taken and sent for analysis to identify the cause of the inflammation, as shown in Table 1. Prednisolone at 1 mg/kg/day was given without clear supportive evidence of the inflammatory cause. However, the clinical outcome became even worse. Despite an improving VA from LP to hand motion (HM), her ocular examinations showed increasing hypopyon to 2.0 mm. Newly developed scleral thinning and melting were also found over the vitreous tapping and intravitreal injection sites (superonasally and superotemporally) with a prolapsed uveal tissue, as shown in Fig. 2. During admission, an oral ulcer at the palatoglossal fold and tonsillar bed was found together with enlargement of the cervical lymph nodes. A cervical lymph node and oral ulcer biopsy was done by an otolaryngologist, with the pathological report revealing ENKTL, as shown in Fig. 3. A computerized tomography scan of the chest, neck, and whole abdomen was done presuming ocular masquerade syndrome with NKTL. Topical and systemic antibiotics were then withdrawn, and a hematologist consultation was done. The patient underwent bone marrow biopsy and aspiration, which showed minimal marrow involvement by ENKTL with scattered Epstein–Barr virus positive cells. NKTL stage IV was diagnosed. Then, preparations were made for the patient to receive chemotherapy via a SMILE regimen [steroid (dexamethasone), methotrexate (MTX), ifosfamide, L-asparaginase, and etoposide]. After her first cycle of chemotherapy (CMT), it was found that her VA in the left eye was LP, there was no progression in the scleral melting, the hypopyon contracted, and the size and amount of iris nodules were decreased, as shown in Fig. 4. Unfortunately, her left eye became phthisis with a complicated mature cataract, 9 months following the diagnosis. Regrettably, after her 5th cycle of CMT, or 2 years after the initial diagnosis, she developed septic shock and died.

**Table 1:** Patient blood investigations

	Value	Reference range	Unit
Complete blood count			
Hemoglobin	11.6	12.0–14.9	g^*^/dl^†^
Hematocrit	36.5	37.0–45.7	%
White blood cell	5.17 × 10^3^	4.4–10.3 × 10^3^	/μl^‡^
Neutrophil	59.2	40.4–73.1	%
Lymphocyte	26.5	20.3–47.9	%
Platelet count	262 × 10^3^	179–435 × 10^3^	/μl
Erythrocyte sedimentation rate	27	0–20	mm/h^§^
C-reactive protein	11.83	< 5.0	mg^II^/l^¶^
Blood urea nitrogen	9.4	6–20	mg/dl
Creatinine	0.53	0.51–0.95	mg/dl
Aspartate aminotransferase	48	0–32	u^**^/l
Alanine aminotransferase	79	0–33	u/l
Fasting blood sugar	88	74–99	mg/dl
Venereal disease research laboratory	non-reactive	non-reactive	
*Treponema pallidum* hemagglutination assay	non-reactive	non-reactive	
Antinuclear antibody	negative	negative	
Rheumatoid factor	4.51	< 4.5: negative, 4.5 - ≤6: borderline, > 6: positive	u/ml^††^
Interferon-gamma for tuberculosis	negative	negative	
Hepatitis profile			
Hepatitis B surface antigen	negative	negative	
Hepatitis C antibody	negative	negative	
HIV antibody test	negative	negative	
Toxoplasma IgG^‡‡^, IgM^§§^	negative	negative	
Hemoculture 2 samples	no growth	no growth	
Mid-stream urine culture	no growth	no growth	
Stool exam	no RBC/ WBC/ parasite		
Antibody to proteinase 3	negative	negative	
Antibody to lactoferrin	negative	negative	
Antibody to myeloperoxidase	negative	negative	
Antibody to elastase	negative	negative	
Antibody to cathepsin G	negative	negative	
Antibody to bactericidal/permeability-increasing protein	negative	negative	
**CD**^¶¶^ **4+ T cell**			
CD 4+ T cell (%)	30.20	24.10–50.70	%
CD 4+ T cell (Absolute count)	540	470–1404	cells/μl
**CD 8+ T cell**			
CD 8+ T cell (%)	35.88	17.10–44.60	%
CD 8+ T cell (Absolute count)	642	360–1250	cells/μl
CD4/CD8 ratio	0.84	0.65–2.49	–

^*^g  gram, ^†^*dl *deciliter, ^‡^*μl *microliter, ^§^*mm/h *millimeter/hour, ^II^*mg *milligram, ^¶^*l *liter, ^**^u = unit, ^††^*ml* = milliliter,

^‡‡^*IgG *immunoglobulin G, ^§§^*IgM *immunoglobulin M, ^¶¶^*CD *cluster of differentiation

## Discussion and conclusions

Our case study involved a rare intraocular presentation of UMS resulting from ENKTL. Its presentation can sometime lead to misdiagnosis and it can be difficult for general ophthalmologists to manage. The first case of intraocular ENKTL was reported by Maruyama et al. in 2015, and involved a 66-year-old female patient with a VA HM. She presented with dense vitreous opacity and vitreous clumping [6]. Takimoto-Shimomura reported a similar presentation of intraocular ENKTL in a 70-year-old woman who presented with severe vitreous opacity. She responded well to IVT MTX, localized irradiation, and a SMILE regimen. She remained relapse-free for 40 months. Her final vision was noted to be better than 20/200 [8]. Zhang et al. reported the case of a 55-year old female ENKTL patient, who initially presented with bilateral panuveitis, retinal and choroidal detachment, and secondary ocular hypertension [2]. Abedi et al. reported the case of an 86-year-old male patient who had a clinical picture of endophthalmitis following intravitreal bevacizumab injection after treatment for diabetic retinopathy. *Streptococcus pneumoniae* grew from his eye discharge. The eviscerated globe revealed high-grade ENKTL [5]. A summary of the reported cases in the literature of intraocular ENKTL in terms of their demographics and characteristics compared to our case is shown in Table 2. Our patient was relatively young compared to the patients in previous reports. She was initially diagnosed with endogenous endophthalmitis, but was not clinically responsive to antibiotics locally and systemically applied. Her immune status was evaluated not only for blood sugar and anti-HIV testing but also for the level of cluster of differentiation 4 and 8, which were found to be normal. Thorough investigations showed no supporting evidence of infectious etiology. Atypical and uncommon infections, such as atypical mycobacteria, fungus, parasite, or even a virus, could not be completely ruled out. Under that circumstance, diagnostic pars plana vitrectomy (PPV) would be extremely helpful. Unfortunately, progressive scleral melting and scleral perforation were a concern in this patient after she developed scleral melting and uveal exposure after the vitreous tapping and intravitreal antibiotic injections. Ultimately, we decided not to perform diagnostic PPV because there was also a worry about the possibility of progressive scleral melting following the surgery. Aqueous tapping was an alternate option since it is a minimally invasive procedure, but the limited amount of aqueous sampling might have interfered with the results. The patient’s unresponsiveness to antibiotics made us think of immune-related inflammatory processes, such as sarcoidosis and granulomatosis with polyangiitis, even though she had only unilateral eye involvement. Without supportive evidence of a specific systemic inflammatory disease, we ultimately decided to prescribe her high-dose prednisolone (1 mg/kg/day). Unfortunately, only one day following the commencement of steroids treatment, her hypopyon level increased, which was an unusual response of the inflammatory process to corticosteroids. This led us to consider masquerade syndrome. Subtle conjunctival injection throughout the treatment period also supported our hypothesis. Diagnostic PPV was then re-considered. The diagnostic yield of PPV in uveitis was previously studied and found to range from 14 to 61.5% [10–12]. According to one retrospective study of 828 patients with uveitis in the Netherlands, 40 patients (5%) were diagnosed with UMS. The vast majority of these (19/40, 48%) had intraocular malignancies and most of them (13/19, 68%) had a lymphoma. Among these, only one patient had UMS originating from T-cells [13]. We are generally familiar with classic presentations of B-cell lymphoma, such as a choroidal mass with or without retinal pigment epithelial alteration, uveal infiltration, or vitreous opacity, but lack experience with intraocular ENKTL. While we were discussing the risks and benefits of performing or not performing PPV with the patient, lymphadenopathy and oral ulcers developed, which were crucial clues finally leading to the precise diagnosis. After the diagnosis was made, we did not perform intravitreal MTX injection or rituximab injection because we decided to observe the patient for her intraocular inflammatory response following systemic CMT. Unfortunately, she developed phthisis bulbi and we believed that performing adjunctive treatment in a phthitic eye would not allow her to be able to regain her vision. Since ENKTL generally has a poor visual outcome, aggressive local therapy may be considered [1, 6, 8]. Delayed diagnosis is a problematic issue in intraocular ENKTL according to its high potential for misdiagnosis. The age of onset cannot completely be used to rule out a differential diagnosis of UMS. Histopathological examination of positive extraocular tissue, like the lymph nodes and oral ulcers in this patient, with multidisciplinary approaches is extremely helpful.

**Table 2:** Demographics and characteristics of intraocular NKTL patients

Reported cases	Age	Sex	Laterality	Previous treatments	VA*		Presentations	Orbital involvement	Pathology/ cytology	Treatment		Death
					Pre	Post				Local	Systemic	
**Present study**	43	F^†^	Unilateral	Antibiotics, steroids	FC^‡^	LP^§^	Hypopyon panuveitis	No	Oral ulcer and cervical lymph node	No	SMILE^II^ regimen	Yes 2 Yr^¶^
**Maruyama et al.** [6]	66	F	Unilateral	Steroids	HM**	NA^††^	Posterior uveitis	No	Retina and vitreous	IVT MTX^‡‡^/ irradia-tion	SMILE regimen	NA
**Tagawa et al.** [7]	50	F	Bilateral	Steroids	6/4.8, 6/12	NA	Posterior uveitis, ERD^§§^, choroidal mass	No	Bone marrow	Had not started the treatment		Yes 1 Mo^¶¶^
**Hughes et al.** [4]	51	M***	Unilateral	Steroids	NA	NA	Panuveitis, ERD, scleritis	No	Nasal turbinate	No	SMILE regimen	NA
**Abedi et al.** [5]	86	M	Unilateral	Antibiotics	NPL^†††^	Evisce-rated	Panuveitis	Yes	Uvea	Evisce-ration	NA	Yes 6 Wk^‡‡‡^
**Okada et al.** [1]	73	F	Bilateral (10 months apart)	Steroids, acyclovir	1st eye 6/30	NA	Anterior uveitis, 2° OHT^§§§^	Yes	Orbital mass	Irradia-tion	DeVIC^¶¶¶^ regimen, intrathecal MTX	Alive at least 14 Mo
					2nd eye 6/12	6/6	Panuveitis	No	Vitreous	No	SMILE regimen	
**Zhang et al.** [2]	55	F	Bilateral	Steroids, ganciclovir	PL****	NA	Panuveitis, ERD, Cd^††††^, 2° OHT	Peri-orbital	Naso-pharynx	No	Yes	Yes 1 Mo
**Takimoto-Shimomura et al.** [8]	70	F	Unilateral	NA	6/60	Improved	Vitritis	No	Vitreous	IVT MTX/ irradiation	Modified SMILE	Alive at least 40 Mo

*VA visual acuity, ^†^*F *female, ^‡^*FC *finger counting, ^§^*LP *light projection, ^II^*SMILE *steroid (dexamethasone), methotrexate, ifosfamide, L-asparaginase, and etoposide, ^¶^*Yr *year, **HM Hand movement, ^††^*NA *not applicable, ^‡‡^*IVT MTX *intravitreal methotrexate, ^§§^ERD exudative retinal detachment, ^¶¶^*Mo *month, ***M male, ^†††^*NPL *no light perception, ^‡‡‡^*Wk *week, ^§§§^*OHT *ocular hypertension, ^¶¶¶^DeVIC dexamethasone, ifosfamide, carboplatin, and etoposide, ****PL perception of light, ^††††^*Cd *choroidal detachment

## Data Availability

The datasets used and/or analyzed during the current study are available from the corresponding author upon a reasonable request.

## Abbreviations

CMT: Chemotherapy
ENKTL: Extranodal Natural Killer/T-cell Lymphoma
HM: Hand motion, hand movement
IVT: Intravitreal
MTX: Methotrexate
LP: Light projection
PPV: Pars plana vitrectomy
SMILE: Steroid, methotrexate, ifosfamide, L-asparaginase and etoposide
UMS: Uveitis masquerade syndrome
VA: Visual acuity
